# Epstein-Barr Virus (EBV)-Associated Myopericarditis Provoking Severe Heart Failure in an Immunocompetent Young Male

**DOI:** 10.7759/cureus.62315

**Published:** 2024-06-13

**Authors:** Edwin R Mosquea Gomez, Vanessa Castellanos, Isaac Soliman, Bijal Mehta

**Affiliations:** 1 Internal Medicine, Hackensack Meridian Mountainside Medical Center, Montclair, USA

**Keywords:** epstein-barr virus, inflammation and heart failure, immunocompetent adult, acute myopericarditis, cardiogenic shock

## Abstract

Myopericarditis, a rare inflammatory condition affecting the heart and its surrounding layers, can lead to serious consequences if not promptly diagnosed and treated. A recent case involved a 28-year-old man with no significant medical history who developed severe chest pain and was diagnosed with myopericarditis induced by the Epstein-Barr virus (EBV). The patient's symptoms, imaging, and lab test results suggest myopericarditis. Initially, he was treated with non-steroidal anti-inflammatory drugs (NSAIDs) and colchicine, and upon discharge, he continued with NSAIDs, as well as guideline-directed medical therapy, including an angiotensin-converting enzyme inhibitor, beta blocker, and SGLT2 inhibitor. Close follow-up with the cardiology and heart failure programs was planned. This case highlights the rare occurrence of this condition in individuals with a healthy immune system.

## Introduction

The heart is a vital organ that is composed histologically of different layers, namely, innermost endocardium, myocardium, and pericardium. Myopericarditis is an inflammatory process of the myocardium and pericardium, which can occur due to infectious processes and may present with a wide range of symptoms, from mild to severe [1-3]. Epstein-Barr virus (EBV)-induced myopericarditis is a rare condition that can lead to cardiogenic shock in immunocompetent patients [4] with an incidence that remains unclear and a diagnosis that is based on history and physical examination, along with electrocardiographic changes, myocardial injury, and serum inflammatory biomarkers [5-7]. Management depends on the severity of the lesion as well as complications that can arise [6,7]. Clinicians should be mindful of the potential for severe presentations, even in immunocompetent patients.

## Case presentation

A 28-year-old male with no past medical history other than undergoing recent rhinoplasty due to a deviated septum presented to the emergency department with crushing left-sided chest pain for few hours which woke him up from his sleep. The chest pain was abrupt and sudden, associated with shortness of breath and profound diaphoresis. The patient adamantly denied any drug use, as well as alcohol intake or smoking history. The patient had recent surgery about two weeks prior to presentation and later he endorsed having some upper respiratory symptoms with cough, malaise, and fatigue, four days prior. He denied any travel, COVID-19 vaccine, prior cardiac history, or any relevant family history.

**Figure 1:**
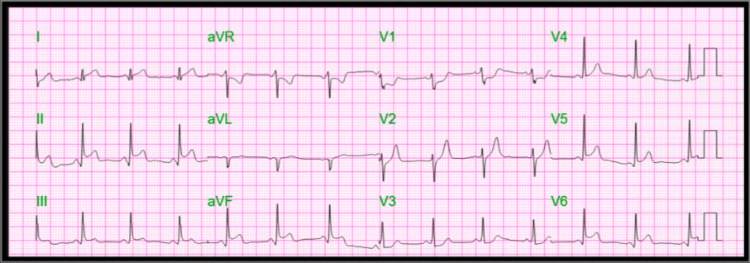
Electrocardiogram showing ST depression V1 and aVR, ST elevation in V5-V6, II, and aVF.

The initial electrocardiogram (ECG) showed ST segment elevation in inferior and anterior leads and aVR, V1-V3 depression consistent with acute myocardial infarction (Figure 1). Laboratory tests showed an elevation of troponin I to 17.6 ng/mL, white blood cell count mildly elevated to 16,000 uL, hemoglobin 13.8 g/dL, mildly elevated transaminase alanine transaminase 71 U/L, aspartate aminotransferase 160 U/L, C-reactive protein elevated to 151 mg/L, and erythrocyte sedimentation rate 96 mm/hr.

**Figure 2:**
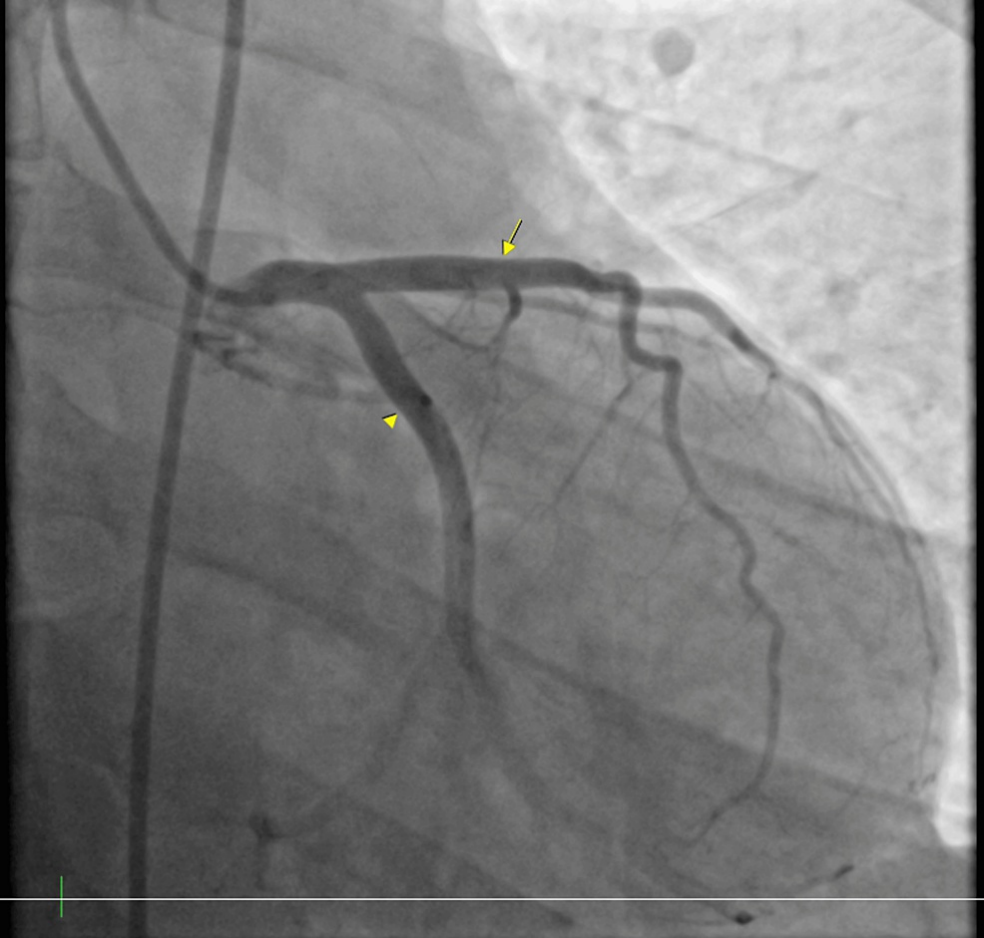
Left heart catheterization showing clean circumflex (arrowhead) and left anterior descending arteries (arrow). Left heart catheterization showing a clean circumflex artery and left anterior descending artery indicating that chest pain is not due to coronary disease.

**Figure 3:**
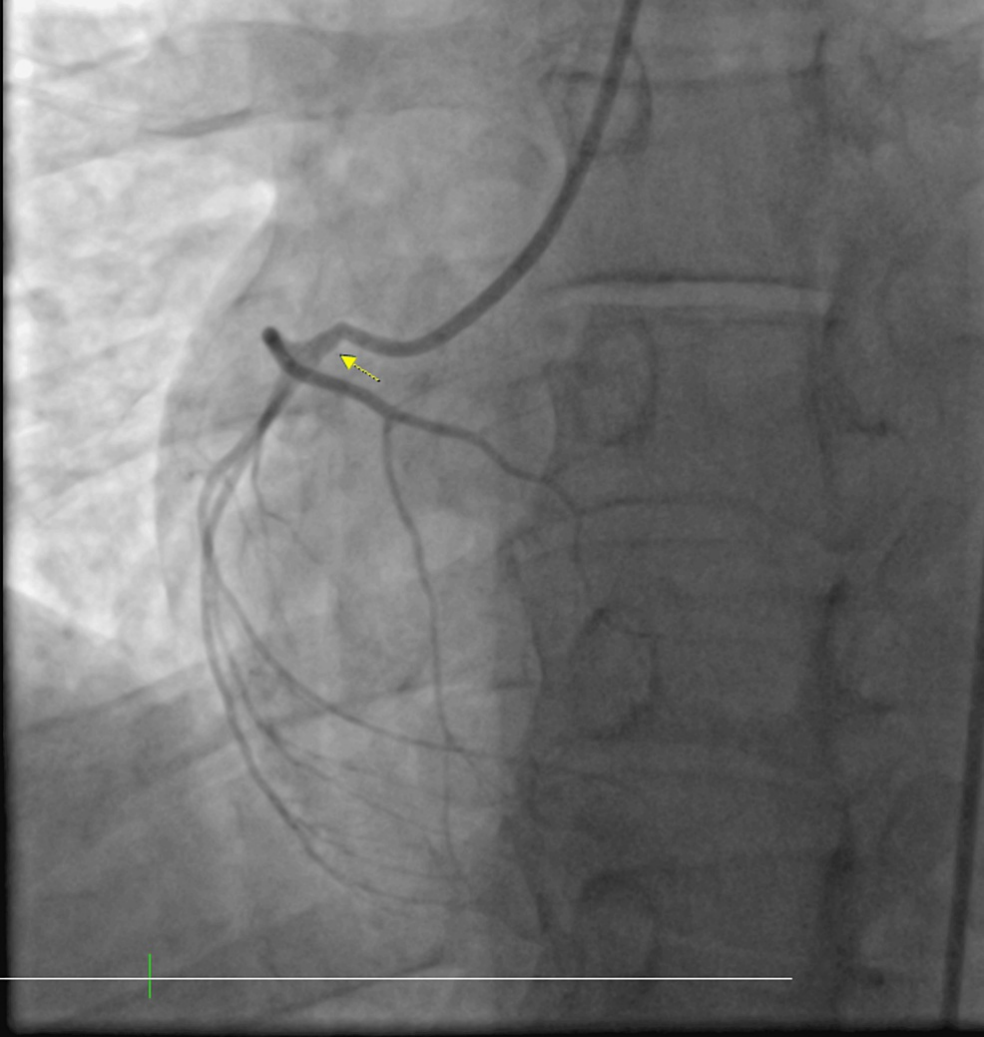
Left heart catheterization showing a clean right coronary artery (arrow). Left heart catheterization showing a clean right coronary artery indicating that chest pain is not due to coronary disease.

**Figure 4:**
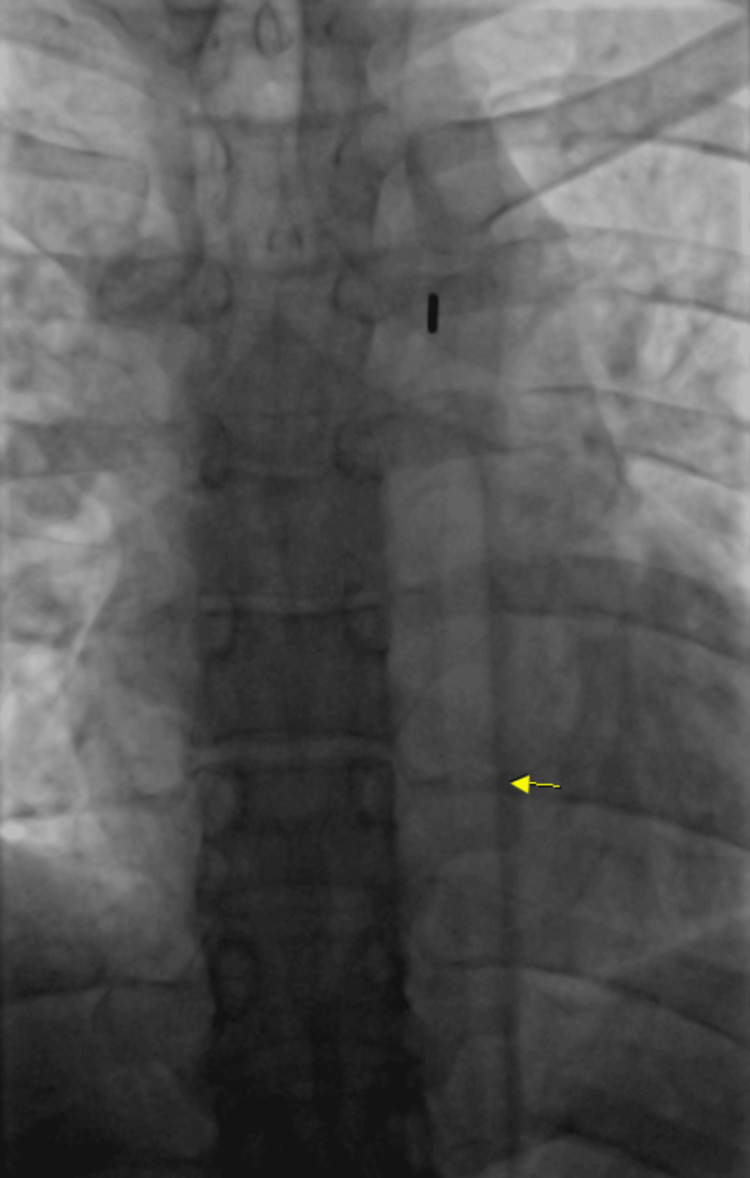
Intra-aortic balloon pump placement. Intra-aortic balloon pump placement placed to assist in the setting of cardiogenic shock.

A code ST-elevation myocardial infarction was activated and he was emergently taken to the catheterization laboratory. During the procedure, there was no stenosis noted within the coronary arteries (Figures 2, 3); however, he was noted to have left ventricular end-diastolic pressure elevated to 40 mmHg, which indicated myocardial dysfunction and impending heart failure. The decision was made to place an intra-aortic balloon pump (IABP) for stabilization and assist with cardiac contractility (Figure 4). The patient was hemodynamically unstable and in cardiogenic shock. The 2D echocardiogram revealed an ejection fraction of 40-45% with global hypokinesis of the left ventricle and no significant valvular pathology.

He remained somewhat hypotensive but with MAP above 65. Throughout his course, he was later stabilized and the IABP was removed. Subsequent blood work revealed elevated EBV by PCR to 481 and the rest of panel by PCR negative (Table 1). As for the rest of the hospital course, troponin I peaked at 35,000 ng/mL (0.000-0.034 ng/mL) with an elevated pro-BNP to 487 pg/mL (<125 pg/mL) indicative of heart failure.

**Table 1:** Viral panel The table shows the viral panel and positive Epstein-Barr virus by PCR, indicating the likelihood cause of myopericarditis. RSV: Respiratory syncytial virus

Virus	Result
Epstein-Barr virus PCR	481 IU/mL
COVID-19	Not detected
RSV	Not detected
Mycoplasma pneumoniae	Not detected
HIV	Non-reactive
Coxsackie	Not detected
Adenovirus PCR	Not detected
Parainfluenza PCR	Not detected
Rhinovirus/Enterovirus PCR	Non-reactive
Influenza A/B PCR	Not detected
Metapneumovirus PCR	Not detected
Parvo B19 PCR	Not detected
Hepatitis Panel	Non-reactive

His acute presentation, imaging, and laboratory test results point toward likely myopericarditis caused by EBV. He was initially treated with non-steroidal anti-inflammatory drugs (NSAIDs) and colchicine and discharged on NSAIDs, as well as guideline-directed medical therapy with an angiotensin-converting enzyme (ACE) inhibitor, beta blocker, and SGLT2-inhibitor with close follow-up with Cardiology and Heart Failure programs.

## Discussion

Myocarditis is an inflammation of the heart muscle that can cause heart failure, sudden death, and dilated cardiomyopathy [1], identified by conventional histology and immunohistochemistry as infiltration of mononuclear cells to the myocardium [2]. Causes of myocarditis can be classified as infectious vs non-infectious such as viruses, bacteria, protozoa, fungi, toxins, and systemic diseases [3].

EBV is a y-herpesvirus that causes infectious mononucleosis and post-transplant lymphoproliferative disorders [4]. It has been associated with inflammatory cardiomyopathy as a consequence of its cytopathic effect on cardiomyocytes, presenting with different phenotypes, these include progressive cardiac dilatation and dysfunction, coronary syndrome X2 with localization of endothelial cells leading to endothelial dysfunction and angina with normal coronaries and an acute infarct-like myocarditis with cardiogenic shock [5-9]. 

Myocarditis due to EBV is very rare in immunocompetent hosts with a mechanism of cardiovascular damage that is still not understood [9], the exact incidence of which remains unclear, and clinical manifestations can range from asymptomatic to sudden death, due to fulminant heart failure to malignant ventricular arrhythmias [6-8]. Clinical manifestations include fatigue, palpitations, chest pain, dyspnea, decreased exercise intolerance, or syncope. Some patients may experience viral prodrome that can include rash, myo-arthralgias, gastrointestinal and respiratory symptoms several days to a few weeks prior to cardiac symptoms [6].

Diagnosis consists of detailed history and physical examination, along with abnormalities noticed on ECG, troponin levels, and non-invasive cardiac imaging [7,10,11]. The etiological diagnosis during the acute phase consists of the detection of virus or its components (PCR, RT-PCR) [10]. Cardiac biomarker, troponin, is elevated in acute/early onset myocarditis, but its prognostic value remains unclear [11]. ECG is an important tool used in the evaluation of cardiac pathologies, changes include sinus tachycardia, ST and T wave changes, atrioventricular and bundle branch block; PR depression and diffuse ST elevation are usually due to pericarditis [6]. 

Echocardiography (echo) is useful to rule out valve diseases and to monitor the progression of disease as well as response to therapy [6,7]. Changes noticed on echo include global ventricular dysfunction, wall thickness, regional motion abnormality, and diastolic dysfunction [6,7]. T-2 weighted cardiac magnetic resonance imaging has emerged as an important tool in diagnosing myocarditis differentiating ischemic vs non-ischemic, as well as monitoring progression [6,7,10,12]. The gold standard is endomyocardial biopsy but is not a routine practice [6,7,13].

Management consists of optimization of heart failure and arrhythmia control [6,7,9] and a prognosis that varies with factors like severity of presentation, location, and timing of treatment [9]. Mechanical support with an IABP or left ventricular assist device may be necessary in severe cases [11]. 

## Conclusions

Myopericarditis is a pathological process that can result in secondary to infectious and non-infectious processes. It can have a wide range of manifestations. EBV-induced myopericarditis is rare and not fully understood. Diagnosis involves various methods, and the prognosis depends on initial symptoms. This case highlights the rare occurrence of EBV-induced myopericarditis, causing an elevation of troponin as well as ECG changes depicting myocardial injury and severe heart failure with elevated left ventricular diastolic pressures, necessitating IABPs due to cardiogenic shock. Clinicians should be aware that even though patients are immunocompetent and have no symptoms and risk factors, infectious agents like EBV could lead to this pathological process with deleterious complications. 
